# Estimating Fish Exploitation and Aquatic Habitat Loss across Diffuse Inland Recreational Fisheries

**DOI:** 10.1371/journal.pone.0121895

**Published:** 2015-04-15

**Authors:** Derrick Tupper de Kerckhove, Charles Kenneth Minns, Cindy Chu

**Affiliations:** Department of Ecology and Evolutionary Biology, University of Toronto, Toronto, Ontario, Canada; Evolutionary Biology Centre (EBC), Uppsala University, SWEDEN

## Abstract

The current state of many freshwater fish stocks worldwide is largely unknown but suspected to be vulnerable to exploitation from recreational fisheries and habitat degradation. Both these factors, combined with complex ecological dynamics and the diffuse nature of inland fisheries could lead to an invisible collapse: the drastic decline in fish stocks without great public or management awareness. In this study we provide a method to address the pervasive knowledge gaps in regional rates of exploitation and habitat degradation, and demonstrate its use in one of North America’s largest and most diffuse recreational freshwater fisheries (Ontario, Canada). We estimated that 1) fish stocks were highly exploited and in apparent danger of collapse in management zones close to large population centres, and 2) fish habitat was under a low but constant threat of degradation at rates comparable to deforestation in Ontario and throughout Canada. These findings confirm some commonly held, but difficult to quantify, beliefs in inland fisheries management but also provide some further insights including 1) large anthropogenic projects greater than one hectare could contribute much more to fish habitat loss on an area basis than the cumulative effect of smaller projects within one year, 2) hooking mortality from catch-and-release fisheries is likely a greater source of mortality than the harvest itself, and 3) in most northern management zones over 50% of the fisheries resources are not yet accessible to anglers. While this model primarily provides a framework to prioritize management decisions and further targeted stock assessments, we note that our regional estimates of fisheries productivity and exploitation were similar to broadscale monitoring efforts by the Province of Ontario. We discuss the policy implications from our results and extending the model to other jurisdictions and countries.

## Introduction

Recreational fisheries are increasingly replacing commercial fishery landings (in value and tonnage) for many inland and coastal species in North America [1,2] and Europe [3], and is similarly becoming a major use of fish resources in Australia, South America and South Africa (e.g. over 10% of the industrialized world’s population participates in recreational fisheries; [4]. However, high fishing pressure and aquatic habitat degradation from other human activities are causing freshwater fish to become one of the most threatened groups of vertebrates worldwide [5]. Given the high economic value of these fish stocks, including recreational values of $US 41.8 billion in the United States in 2011 [6], £1.18 billion in England in 2005 [7], and $CDN 8.8 billion in Canada in 2010 [8], ensuring the sustainability of these stocks under exploitation and habitat loss should be a priority not only among fisheries managers and the outdoor recreation industry, but also by governments looking to preserve a sustainable economy.

The sustainability of fish stocks under a recreational fishery has only recently been widely questioned and is generally not well understood [3, 9–11]. In 2002, the concept of the invisible collapse of North America’s inland recreational fisheries was presented with evidence of four fish stock collapses which had received little management or public attention [12]. A root cause of this knowledge gap is that the often complex ecological dynamics of fish populations are difficult to study across diffuse inland stocks, which are either too spread out over large management jurisdictions to efficiently monitor, or conversely, partitioned among too many individual fishing clubs with private claims to small sections of the landscape (North America—[12]; Europe—[3]). Both these conditions contribute to low rates of catch reporting to central management authorities [9] and present a challenge in predicting harvest rates. Further, a lack of robust knowledge of the existing and the pre-industrial baseline sizes of inland fish stocks over large spatial scales [3, 13–14] makes it difficult to estimate exploitation levels even if catch rates can occasionally be obtained. Lewin and colleagues [10] present a list of exploitation rates for common inland species which generally range from 10% to 80%, however most of these estimates, as well as typical creel reports [15], are from individual lakes or rivers, and so while substantial, give little insight into general rates of exploitation across large landscapes.

Habitat degradation has not been well addressed alongside the scenarios of the invisible collapse and the rates of fish habitat loss are not well known across diffuse aquatic landscapes [5, 16–18]. In consequence, the interaction between fish habitat loss and the recreational fishery is difficult to predict. There are very few theoretical studies that include both habitat loss and harvesting on dynamic consumer-resource systems or metapopulations [19, 20]. However, the few available suggest that habitat driven mechanisms can further obscure the true status of recreational stocks and thus contribute to the invisible collapse scenarios. From a policy perspective, the important role of fish habitat in sustaining healthy fish stocks is increasingly being recognized [10] and is currently the guiding principle of fisheries management under the 2011 *NOAA Habitat Blueprint* in the United States [21] and the European Union *Habitats Directives* [20]. However, conflicts between land-use and fisheries management objectives can still occur when policy makers, fisheries managers, scientists and the public weigh the known economic value of a development against the unknown, conceptual value of fish habitat (United States—[22]; Europe—[3]; Canada—[23]). Thus, understanding the rates of habitat degradation in active fisheries is an important first step in ascribing a known value to fish habitat.

In an effort to address the pervasive knowledge gaps in the state of recreational fisheries, this study demonstrates a method to estimate indicators of exploitation and aquatic habitat degradation across diffuse fisheries. First, we use landscape inventories of aquatic resources to estimate rates of fishable production in terms of theoretical sustainable yields in lakes and rivers that are accessible to anglers [24]. Second, we use a regional creel survey of anglers’ catch to characterize exploitation rates as the percentage of the fishable production remaining after the anglers’ harvest (i.e. the difference between the sustainable yield and harvest, as a percentage of the sustainable yield). Third, using applications for development permits and audits of environmental compliance, we estimate the area of aquatic habitat that was ideally protected per year, as an indicator of the amount of fish habitat threatened each year by human activities.

We demonstrate our approach with one of the largest managed inland recreational fisheries in North America (Ontario, Canada), but it can be extended to other countries to evaluate recreational fisheries across large spatial scales. We argue that the spatial aspects of recreational fisheries are critically important considerations because a) anglers are often mobile and to a certain extent are expected to maximize fishing opportunities across multiple watersheds and jurisdictional boundaries [25, 26], b) fish stocks and fish habitat are often defined from a management and ecological perspective across broad spatial scales [27], and c) the interaction between high harvests and aquatic habitat degradation is primarily a spatial question of how and where these two pressures overlap [28]. Ontario provides a good test for our approach because Canadian federal and provincial governments do not actively track rates of aquatic habitat loss and degradation [29] and the management of the recreational fishery has only recently been switched from a traditional population-centric approach to a broad-scale monitoring program [30]. Further, Ontario has experienced documented collapses of recreational fisheries such as the declines of Lake Trout and Lake Whitefish in the 1950s and 1960s, respectively, in Lake Simcoe [31] and started maintaining the production of over 60% of the natural Lake Trout populations with stocking in the 20^th^ Century [32].

Landscape approaches to fisheries and aquatic environments management are increasingly found across the world in response to the challenge of managing a wide network of diffuse lakes and watersheds with limited human resources. John Downing’s 2013 Presidential address to the Association for the Sciences of Limnology and Oceanography identified regional approaches to inland waters as the top research paradigm to emerge in the next 10 years [33]. Classification systems for identifying areas of concern across broad aquatic landscapes are increasingly being prioritized by governments [34], and there is a growing recognition that cumulative effects may only be properly managed at large landscape scales [35]. Our method follows this important trend by gaining insight into the status of a fishery by presenting a baseline of fish resources in the same spatial perspective as the threats to habitat loss. By presenting regional estimates in this manner, fisheries managers can prioritize areas requiring greater monitoring or management effort; much like the relative disturbance index for US watersheds [35]. Thus our goal is to provide a “triage” method for identifying vulnerable watersheds or management zones, as well contribute a broad spatial picture of the health of a fishery.

## Materials and Methods

### Methodological Theory

This model allows rates of exploitation and habitat loss to be estimated and displayed on a regional basis so that managers can prioritize areas that have a high potential to be vulnerable to fishery declines, and target resources for management actions. It is not a stock assessment model, yet it provides reasonable estimates of regional rates. The methodology involves two main predictions: 1) a baseline level of fish productivity which can vary depending on the size and location of the aquatic habitat and 2) the area of aquatic habitat potentially affected by human activity or climatic conditions. A baseline level of fish productivity that represents the potential fish productivity for a region can be calculated using regional models of sustainable yield [13], scaling up from primary productivity [14] or using land-use regression models (shown here). The area of aquatic habitat lost can be calculated by determining the statistical distribution of impact sizes associated with a particular activity, and sampling from that distribution for each development initiated on that year of study. In addition, if environmental regulations are in place to mitigate or compensate for the development’s impacts, the rates of efficacy of the environmental practices can be applied to estimate the ultimate impact size of the development [36]. This method requires at a minimum 1) fish harvest or catch information from the recreational fishery, 2) landscape information for the sizes, depths and locations of aquatic habitat, 3) current or historical climatic variables to calibrate productivity estimates, 4) numbers and types of new industrial, residential, commercial or infrastructure developments, 5) extent of road networks or a general estimate of the accessibility of aquatic habitats, and 6) environmental audits of the ecological impacts of human activities. The method is demonstrated in context with our study area, but could easily be developed elsewhere. Note that additional methodological detail and associated data tables for Ontario can be found in S1 File.

### Study Area Regulatory Landscape

The fisheries within the province of Ontario are managed at the provincial level by the Ontario Ministry of Natural Resources and Forestry (MNRF). Fish habitat, on the other hand, falls under federal jurisdiction and is managed by the federal Department of Fisheries and Oceans Canada (DFO). This study’s results were reported using the MNRF’s Fisheries Management Zones (FMZs) as the smallest spatial unit; FMZs are based on watershed and logistically convenient boundaries (see Figure A in S1 File), and all fall within one federal management zone (DFO’s Central and Arctic Region). Note that FMZ 12, Pembroke/Ottawa River, is a very small management zone for which the classification of the aquatic environment is ambiguous because of the size of the Ottawa River, and was excluded from the study.

### Lake Productivity Estimate

Limnological parameters (e.g. mean depth, total dissolved solids) were acquired for a set of lake size classes within secondary watershed units with surface areas less than 100 km^2^ from the Canadian Lakes Assessment Model [37] and from individual lake surveys for larger waterbodies [38]. Fishable productivity was estimated for these lakes using the quadratic form of the 1982 sustainable yield model of Schlesinger and Regier [13] which is based on the morpho-edaphic index (MEI; a function of total dissolved solids and mean depth) and mean annual air temperature (MAATC; taken from 1971–2000 Canadian climate normals data). The 1982 dataset from Schlesinger and Regier [13] includes Ontario lakes and so was re-analyzed using multiple regression of ln(MEI), ln(MEI)^2^ and MAATC on ln(Yields). Although criticized in the latter 20^th^ Century, the MEI in general, and the specific use of lake morphometrics and nutrients (i.e. Phosphorus, total dissolved solids), has gained wider acceptance as a robust correlate to fish and aquatic production [39–41]. However, it is based on the productivity of commercial yields and so we tested how easily the relationship could be extended to recreational yields derived from two creel datasets across Ontario [15, 42] with our predicted values using the Root Mean Square Error between the paired estimates. The secondary watersheds and large lakes were apportioned within the Ontario FMZs boundaries in ArcGIS 9.2 (Environmental Systems Research Institute Inc., Redlands, California, USA) so that lake and fishable productivity estimates would be FMZ specific. The Great Lakes were only considered up to the Ontario border. Last, a stress index was applied to the fishable productivity to include the expected changes in habitat quality leading up to the study year (i.e. fish yields developed from an MEI model based mostly on pristine lakes in the 1960s and 1970s will be affected by change in the climate and other anthropogenic disturbances). The stress index values were derived in previous studies [16, 27] and were directly applied to weight the yield values for the study year to generate the FMZ-specific fisheries production estimates.

### River Productivity Estimates

River area and biomass density were used to estimate riverine productivity in Ontario. Total lengths of the rivers within each FMZ were summed using the Ontario Hydrometric Network (OHN) Watercourse layer for which the total polyline lengths within each FMZ were multiplied by the available spatial resolutions (i.e. 10, 20 or 50 m) to estimate river areas. Larger river segments (generally > 50 m wide) were identified using the OHN Waterbody layer. Areas for all of the rivers were summed in ArcGIS 9.2 to produce total river area estimates for each FMZ. A stepwise multiple linear regression model was developed to estimate average river fish biomass density (kg·ha^-1^) at the tertiary watershed scale [43] from climate parameters [44] and landscape variables (see regression parameters in Table A in S1 File). Observed average biomass density of fish in 28 watersheds was provided by the MNRF HabProgs database [45] and MNRF Scientists (see Table B in S1 File). Using the most parsimonious regression model (model in Table C in S1 File), average biomass densities (*B*) were estimated for each tertiary watershed in Ontario. A standard production-biomass relationship [46] was used to predict average biomass production (*P*: kg·ha^-1^·yr^-1^) as *log*_*10*_ *P = 0*.*38 + 0*.*89 log*_*10*_ *B*.

*P* represents the total fish production of the river, and is therefore not equivalent to the fishable production estimates derived from the lake fishery yields. Therefore, we calculated the proportion of biomass in each of the 28 watersheds in our database that was derived from fish known to be targeted by anglers (see Table D in S1 File for their species names and average weights taken from creel studies across Ontario). Multiplying this proportion by *P* gave us an estimate of production that could be harvested by anglers in one year i.e., the fishable production. Area-weighted average fishable production in each of the FMZs was then calculated from the tertiary watershed and FMZ layers using zonal geostatistics in ArcGIS. Total fishable production estimates for each FMZ were calculated as the average fishable biomass production multiplied by the riverine area of each FMZ. Note that a stress index was not required as the fish biomasses were those observed under recent conditions.

### Accessible Fishery Estimates

Not all the fish production in Ontario is readily accessible because Ontarian anglers are unlikely to hike more than a kilometer from an established road to access a waterbody [26]. To estimate the lakes and rivers directly accessible to the recreational fishery we overlaid the Ontario Road Network shape file in ArcGIS (accuracy to 10 m), buffered the spatial extent of the roads by 1 km on either side, and recalculated fishable production for only the proportion of the FMZ that was connected to the buffered road network. Lakes within 1 km of a road were treated as accessible whereas river road crossings within 1 km of a road provided access to the entire tertiary watershed. The latter condition assuming that boat travel will facilitate access throughout the watershed systems. While this assumption could be violated for some species, over 85% of the riverine fishes caught by Ontario anglers in 2005 completed watershed-wide migrations [47]. Therefore, these fishes could be accessible to the fishery in the absence of instream barriers to movement.

### Fishable Production Surplus Estimates

Angler catch-only and harvest data are reported by FMZ in the MNR 2005 Ontario Angler Survey [47]. This survey is completed by mail, and verified for accuracy by comparing it with creel surveys [48]. In the evaluation of the 2005 Angler Survey harvest rates were expected to be up to 2 or 3 fold higher than creel estimates yet well correlated with the creel. However, as creel estimates general underestimate harvest rates it was difficult to determine which value to use. In the recent 2010 Angler Survey (not released) the 2005 catch and harvest data was corrected by decreasing the values by 13.85% for residents and 8.9% for non-residents (pers. comm. Dr. Helen Ball, MNRF). As these corrections did not qualitatively change our conclusions (i.e. none of the over-exploited FMZs became sustainably harvested) and the 2010 Angler Survey was not widely available at the time of the analysis we did not apply the corrections, yet list them here as a caveat to our reported surplus yield values. Catch-only and harvest data are reported in fish numbers however, using the MNRF’s extensive active creel survey database (i.e. data collected on site) we were able to assign average weights per fish species caught in the province (see Table D in S1 File).

The tonnage of fish tissue removed annually from Ontario waters should include the harvested fish and the portion of catch-only fish that die upon release. Hooking mortality rates from catch and release fisheries are not well known yet expected to range from 0 to 80% [49,50] depending on the species, ambient water temperature and angling method. In light of the great variability in rates we applied a conservative estimate of 10% hooking mortality to catch-only fisheries. We calculated theoretical exploitation levels as a yield surplus estimate [24]: the difference between the total weight of fish tissue removed (*H*) and the total theoretical annual fishable production (*P*) as a percentage of *P* per FMZ (i.e. *E = (P-H)/P*). We estimated whether the FMZ fishery appeared to be overexploited by how much of the theoretical annual fishable production remained after each year, however if the harvest (*H*) is greater than the theoretical production (*P*) than we report it simply as 0% remaining.

### Habitat Protection Rate Estimates

Our study examined the potential impacts of development projects on physical habitat only and did not include chronic habitat degradation that may be associated with non-point source pollution, permanent changes in land use or climate change. As other studies have examined these more general rates [16,27] we focussed here on those within the regulatory reach of Canadian aquatic managers, and thus the rates of habitat protection within the recreational fishery. These rates are nonetheless unknown [29], and represent the direct influence of human development over or adjacent to aquatic habitat in Canada.

Prior to regulatory changes in 2012 [23], projects which had the potential to impact fish habitat were allowed to proceed under the federal *Fisheries Act (1985)* as long as, 1) Letters of Advice (or Operational Statements) issued by DFO containing directives to mitigate impacts were followed, or 2) the harmful destruction, disturbance or alteration (HADD) of fish habitat was authorized usually as long as habitat compensation projects contributed to an overall no-net-loss of fish habitat [18,51]. We estimated the potential rates of habitat protection by examining all the permits approved by DFO in 2005 in Ontario (see list of all permits in S1 Dataset). The location of the project was identified on the permit by township, or the nearest settlement or waterbody. Using the online Ontario Geonames Database [52], each permit was assigned a latitude and longitude, and was spatially referenced within an FMZ. Further information given included 1) the aquatic habitat type (e.g. riverine or lacustrine), 2) the type of project being permitted identified by Industry, and the Main and Sub Categories of the type of development (e.g. Transportation—Instream Works—Debris Removal), and 3) the type of permit (e.g. Letters of Advice, HADD authorizations). The areas of impacted fish habitat associated with each of these categories could not be provided by DFO and so we had to independently estimate how much fish habitat was protected by each permit. Using data collected from federal audits of environmental compliance under the *Fisheries Act (1985)* [53–56], the frequency distributions of potential impact sizes pertaining to aquatic habitat (e.g. river), project type (e.g. watercourse crossing) and regulatory decision (e.g. Letter of Advice) were modelled using maximum likelihood non-parametric regression methods in the R programming language (R v2.13.1 R Foundation for Statistical Computing 2011) with the MASS and VGAM packages. Seventeen impact distributions were derived for which each had long tails towards rare but extreme values, and were well approximated by Log Normal or ParetoIV equations (see Table E in S1 File). To estimate the 2005 total area of habitat ideally protected per FMZ, 1) each of the 2005 permit entries was assigned an area of habitat by randomly sampling from the appropriate frequency distributions and, 2) the protected area from all permits within each FMZ were summed. The annual rate of habitat protection for 2005 was estimated as the quotient of the total habitat from the summed permits by the total habitat area accessible to the recreational fishery.

Letters of Advice greatly limit the scope of activities within aquatic habitats by guiding developers towards project designs that avoid the need for environmental authorizations. For example, if pipeline crossings are installed when the creek is frozen, a larger HADD typical of pipeline crossings is avoided, and a Letter of Advice is issued instead. Therefore, the habitat areas that Letters of Advice protect can either be small projects or large project specific HADDs. Rather than estimate what proportion of Letters of Advice represent one of these two scenarios, we use them as reasonable lower (Scenario 1) and upper (Scenario 2) bounds to our estimated rates of habitat protection in Ontario. Thus for the lower bound, a general distribution of impact areas for small projects is applied to every Letter of Advice. For the upper bound, project specific HADD frequency distributions are applied to every Letter of Advice except those for mines and hydroelectric projects, whose HADDs are likely too large to merit consideration under this scenario, so instead a general HADD frequency distribution is applied to those project types.

## Results

### Lake Productivity Estimate

The re-analysis of Schlesinger and Regier’s 1982 [13] data led to a significant relationship between the morpho-edaphic index (MEI) and mean annual air temperature (MAATC) with yield (Y; n = 39, Adj. R^2^ = 0.80, p < 0.05) as *ln Y = 0*.*6 + 0*.*13 ln MEI + 0*.*04 ln MEI* ^*2*^ *+0*.*11 MAATC*. Testing this equation against recreational yield data led to a Root Mean Square Error of 3.03 and over-estimates at small yield sizes (See Figure B in S1 File) but generally appropriate values. It is important to remember that recreational yields are not necessarily maximized [13, 42], so it is not surprising that a model based on commercial yields will generate higher estimates. In our approach it is much more important that yields are slightly over-estimated than under-estimate or else the indicators of exploitation rates might always appear too high.

Across the 20 FMZs in Ontario, the proportion of surface area that is covered by lakes range from 3% to 41%, apart from the Laurentian Great Lakes which are closer to 100% but also contain islands. Fishable productivity estimates and annual biomass ranged from 1.97 to 14.72 kg·ha^-1^·yr^-1^ and 230 to 11,555 tonnes· yr^-1^ (see Table 1 and Table F in S1 File). Most of the lakes in southern Ontario are within 1 km from a road, so accessible fishable production only changed dramatically for the north. Less than 5% of the total fishable production was accessible by recreational anglers in the far northwest FMZs.

**Table 1:** Aquatic area, the percentage of habitat protected under the *Fisheries Act (1985)* using the lower bounds of our estimates, the annual fishable production (FP), and the percentage of fishable production remaining after all mortality (harvest and hooking) from the recreational fishery (surplus FP) for lakes and rivers found in Ontario’s Fisheries Management Zones in 2005.

Ontario Fisheries Management Zone	Lake				River			
	Area (km^2^)	Habitat Protected (%)	FP (tonnes)	FP Surplus (%)	Area (km^2^)	Habitat Protected (%)	FP (tonnes)	FP Surplus (%)
1. Far North—Hudson Bay Lowlands	4761	0.00000	2655	100	1006	0.00001	538	100
2. Far North—West	19217	0.00000	8867	50	2291	0.00001	1134	97
3. Far North—East	4885	0.00000	3031	100	1381	0.00763	901	99
4. Red Lake/Sioux Lookout	11858	0.00021	3779	0	1091	0.00005	559	62
5. Fort Frances / Lake of the Woods	12892	0.00004	4036	27	410	0.00019	203	17
6. Thunder Bay	6974	0.00001	1298	40	425	0.00051	243	76
7. Wawa and Nipigon	2644	0.00001	1093	0	662	0.00031	252	83
8. Kirkland Lake	3225	0.00106	1428	14	1521	0.00024	793	87
9. Lake Superior	29035	0.00000	4213	96	35	0.00067	16	100
10. Sudbury/Sault Ste. Marie/Manitoulin I.	3362	0.00010	960	0	565	0.00191	171	41
11. North Bay	2247	0.00010	727	0	299	0.02724	136	0
13. Lake Huron	2622	0.00028	1558	80	0	0.00000	0	100
14. Georgian Bay/North Channel	2775	0.00034	1648	70	18	0.00339	10	0
15. Bancroft/Algonquin	1909	0.00129	716	0	366	0.00268	290	61
16. Guelph (including Lake Simcoe)	1434	0.00039	637	0	588	0.12022	717	0
17. Kawartha Lakes	370	0.01125	231	0	167	0.01163	144	0
18. Eastern Ontario	1316	0.00009	611	0	385	0.01900	347	59
19. Lake Erie/Lake St. Clair/Lower Niagara R.	13609	0.00001	11556	91	36	0.00031	49	0
20. Lake Ontario/St. Lawrence R./Upper Niagara R.	11431	0.00001	6378	91	21	0.06789	21	0

Note: FMZ 12 was not included in the analysis.

### River Productivity Estimates

Sums of the river surface areas cover roughly 2% of Ontario. Across the tertiary watersheds, the measured fish biomass densities ranged from 7.5 to 76.9 kg·ha^-1^. Fish biomass was positively related to mean annual air temperature and negatively related to the proportion of the watershed covered in settlement and developed land (n = 14, Adj. R^2^ = 0.72, p < 0.05). Validation of the model using the data available for 14 additional watersheds had a Root Mean Square Error of 1.08 (see Table C and Figure C, both in S1 File). Average production and total biomass estimates in the FMZs ranged from 24.7 to 101.8 kg·ha^-1^·yr^-1^ and 1,143 to 9,466 tonnes·yr^-1^, respectively (see Table 1 and Table G in S1 File). The average biomass of the species targeted by the recreational fishery equalled 61% of the total species biomass in the well-sampled watersheds. Productivity and total biomass estimates of the species recruited to the fisheries in the FMZs ranged from 15.5 to 59.8 kg·ha^-1^·yr^-1^ and 868 to 5,679 tonnes·yr^-1^, respectively. Similarly to lakes, only rivers in northern Ontario were not connected to roads, however, in our effort to be conservative and include as much production as possible, we’ve likely overestimated how much is actually accessible to the anglers (Table 1).

### Fishable Production Surplus Estimates

**Fig 1:**
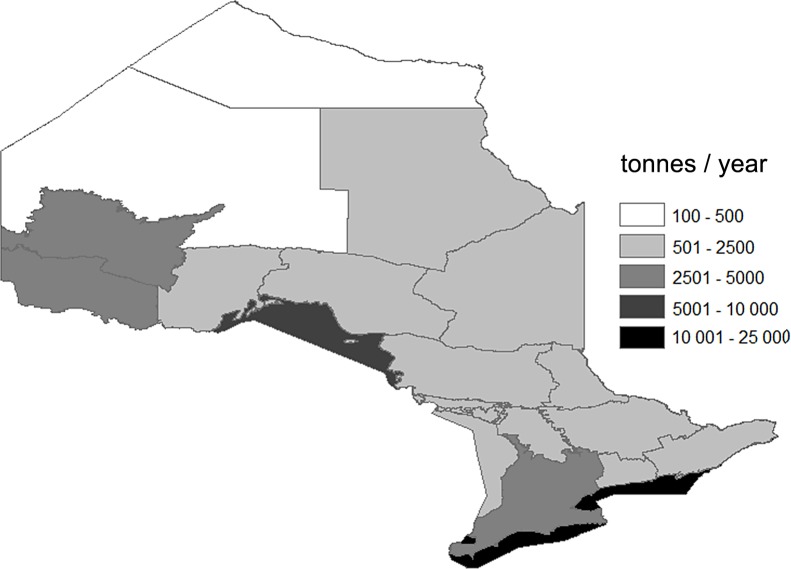
The spatial distribution of the yields of accessible fisheries in 2005 delineated by Ontario’s Fisheries Management Zones.

**Fig 2:**
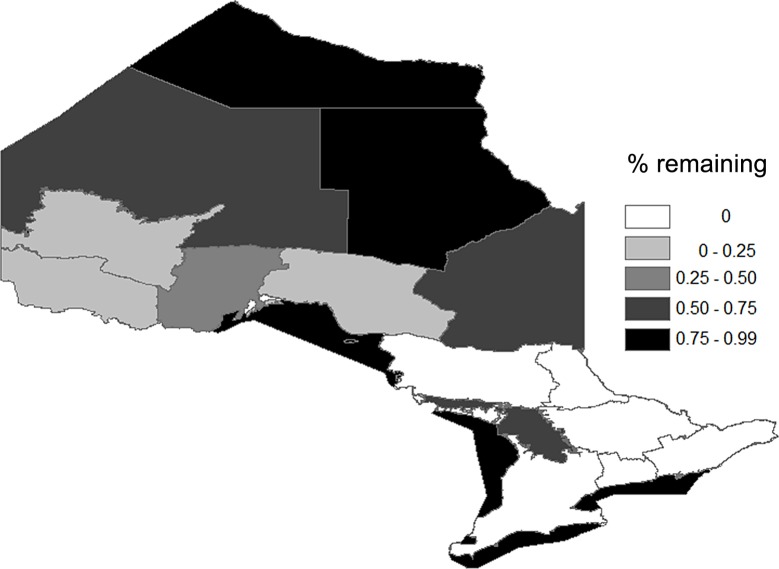
The spatial distribution of the surplus yield remaining following a fishing season in 2005 delineated by Ontario’s Fisheries Management Zones.

About 36 000 tonnes of fish tissue was estimated to be available for sustainable harvesting from the inland FMZs, with another 25 000 tonnes available from the Great Lakes (Fig. 1). The 2005 MNRF angler survey estimated that about 72 000 tonnes of fish were caught and from these catches 14 000 tonnes of fish tissue was harvested. Eighty-five percent of the catch was conducted within the inland FMZs. We estimated that a 10% hooking mortality on catch-and-release fisheries would kill about an additional 6000 tonnes (see Table 2). This substantial addition of fish tissue led to the surplus fishable production being overharvested in all six southern FMZs, whereas without it only two were estimated to be over-harvested (Table 1). All the Great Lakes retained more than 75% of their estimated fishable production (except for Georgian Bay which is delineated separately from Lake Huron and retained 65%). Harvests in the northern FMZs appeared more sustainable however Wawa/Nipigon and Red Lake/Sioux Lookout had less than 20% of the theoretical fishable production remaining (Fig. 2).

**Table 2:** The remaining percentage of the fishable production surplus following the annual fishery in both lakes and rivers under different conditions: 1) FP Alone—fishable production calculated from the entire watershed, 2) Access—only includes the fishable production accessible by the fishery, 3) Hooking—fishable production from the entire watershed but with an additional 10% mortality due to hooking mortality in the catch and release fishery added to the harvest, and 4) All—access and hooking applied to the surplus fishable production.

Ontario Fisheries Management Zone	FMZ Total Surplus			
	FP Alone	Access	Hooking	All
1. Far North—Hudson Bay Lowlands	100	100	100	100
2. Far North—West	97	87	91	66
3. Far North—East	100	100	97	95
4. Red Lake/Sioux Lookout	72	55	46	13
5. Fort Frances / Lake of the Woods	66	56	41	23
6. Thunder Bay	68	63	53	46
7. Wawa and Nipigon	69	41	56	15
8. Kirkland Lake	77	65	66	48
9. Lake Superior	96	96	98	98
10. Sudbury/Sault Ste. Marie/Manitoulin I.	19	7	0	0
11. North Bay	23	15	0	0
13. Lake Huron	83	83	82	82
14. Georgian Bay/North Channel	72	73	69	69
15. Bancroft/Algonquin	36	25	10	0
16. Guelph (including Lake Simcoe)	33	33	0	0
17. Kawartha Lakes	0	0	0	0
18. Eastern Ontario	28	20	5	0
19. Lake Erie/Lake St. Clair/Lower Niagara R.	92	92	89	89
20. Lake Ontario/St. Lawrence R./Upper Niagara R.	87	87	82	82

### Habitat Protection Rate Estimates

**Fig 3:**
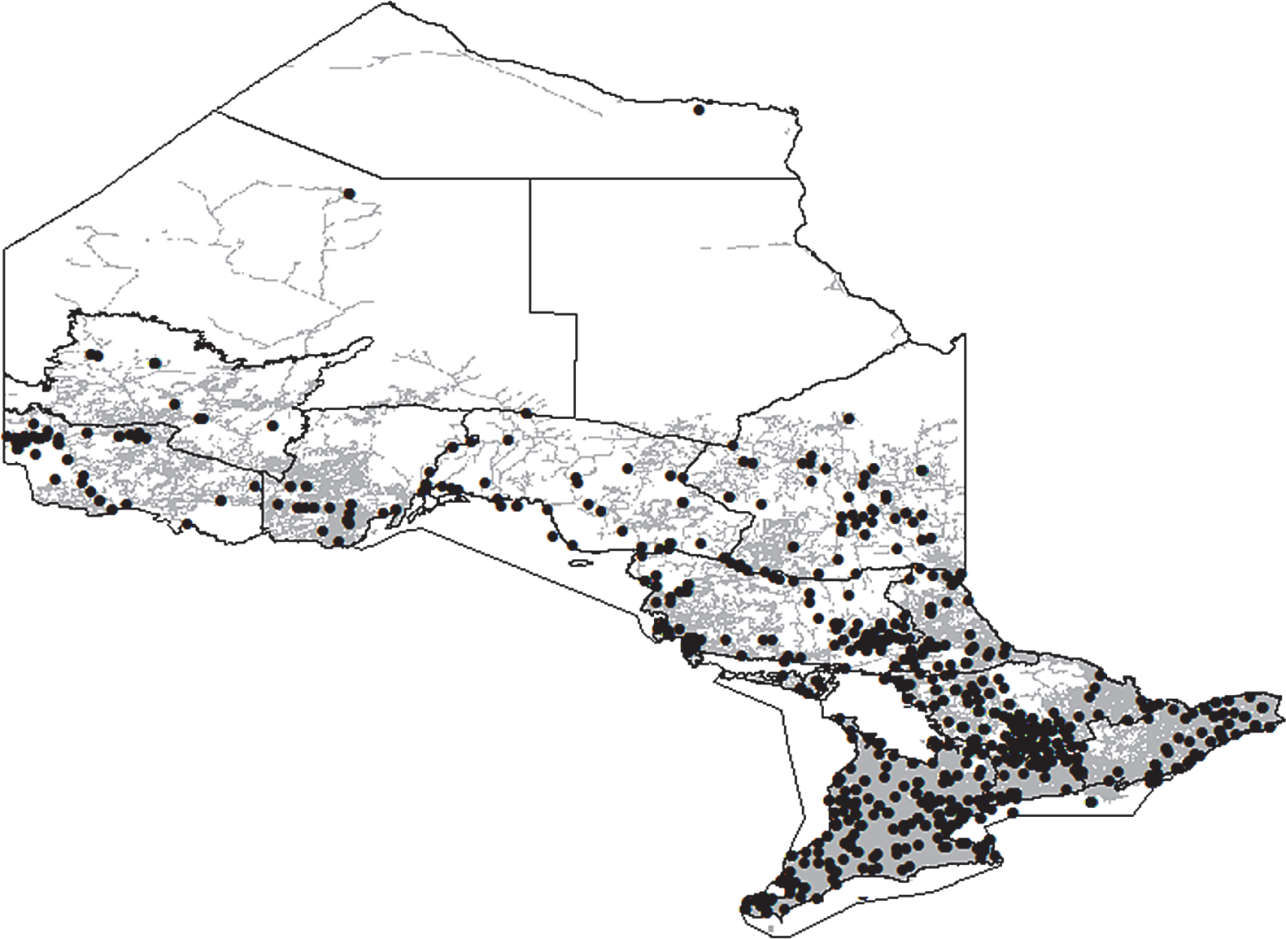
The spatial distribution of the road coverage (grey lines) and distribution of projects (black dots) approved under the *Fisheries Act (1985)* delineated by Ontario’s Fisheries Management Zones.

**Fig 4:**
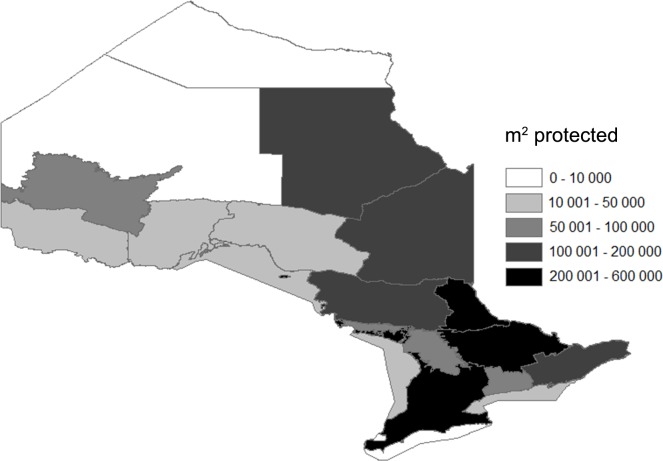
The proportion of individual project sizes in numbers (left column) and in their cumulative sum of potential impact area to fish habitat (right column) from all the permits under the *Fisheries Act (1985)* granted in Ontario in 2005 to developers. We present two scenarios that represent a lower and upper bound to our estimates of fish habitat protected for each permit: Scenario 1) Letters of Advice represent generally small projects, and Scenario 2) Letters of Advice represent projects that without the *Fisheries Act (1985)* would have impacted standard project-specific areas of fish habitat (i.e. HADDs).

**Fig 5:**
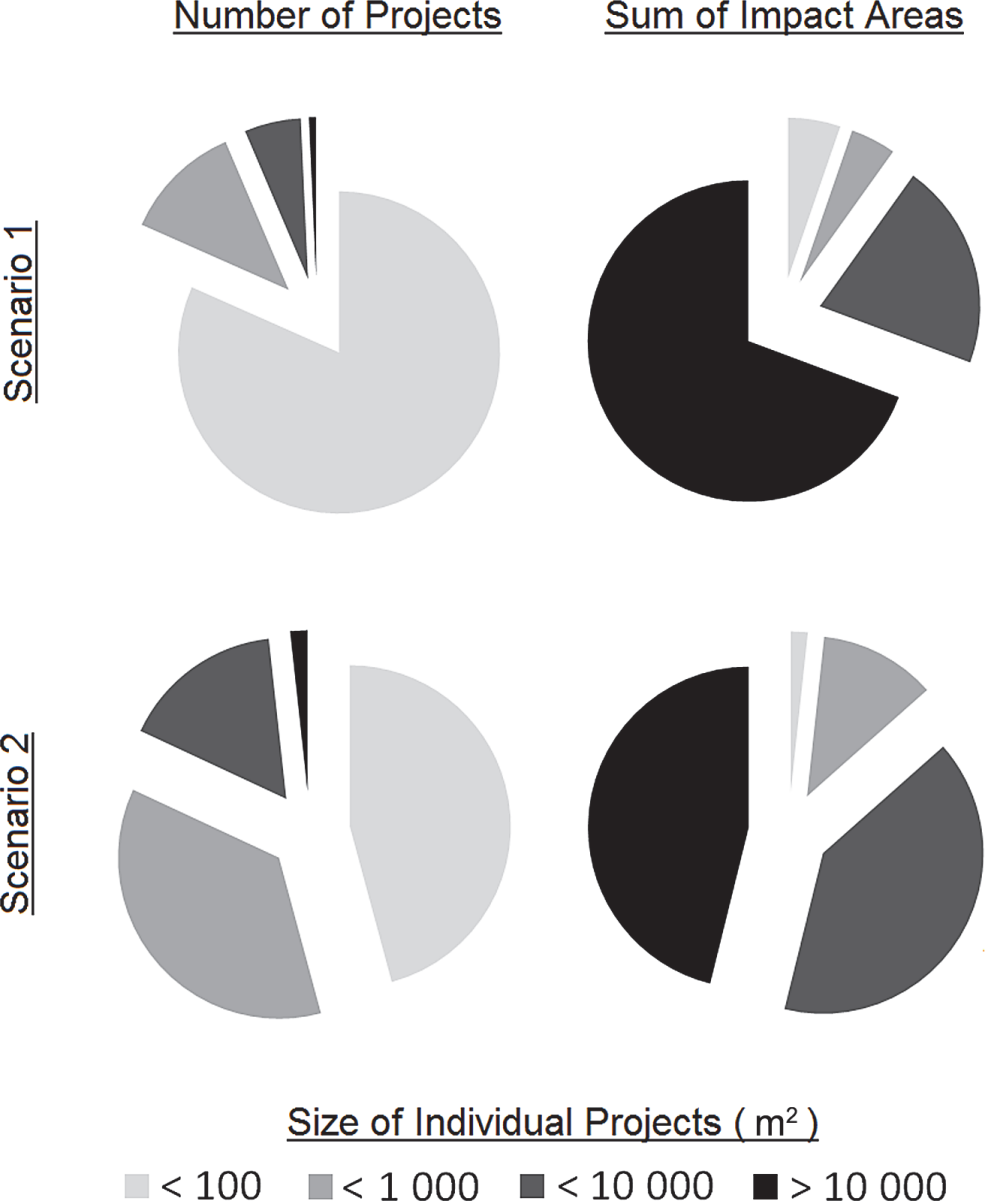
The spatial distribution of the area of fish habitat protected using the average of the lower and upper bounds of our estimates in 2005 delineated by Ontario’s Fisheries Management Zones.

In 2005, 1754 approvals under the *Fisheries Act (1985)* were granted in Ontario across all the FMZs. These approvals were categorized into 436 different types of developments depending on the aquatic habitat, the industry and the specific type of activity. The number of impacts were roughly even between lakes (N = 860) and rivers (N = 814) and much lower for other habitats including 3 estuaries, 26 wetlands, 12 considered not fish habitat (and thus excluded from the analysis) and 39 yet to be properly classified. The majority of the projects approved were found in southern Ontario and within 1 km of an established road (Fig. 3). Between 1.1 and 2.6 km^2^ of fish habitat across the province was estimated to be protected in 2005 with the most common habitat impacts from water management projects, shoreline works on rivers and lakes, and infilling on rivers and lakes. Under both the lower (Scenario 1) and upper (Scenario 2) bounds of our estimates, small projects (i.e. < 1000 m^2^) accounted for over 80% of the total projects but less than 15% of the total habitat protected (Fig. 4). The greatest impacts to fish habitat were estimated to be contributed by a very small proportion (i.e. < 2%) of very large projects (i.e. individually > 100 000 m^2^). In comparison to Ontario’s accessible aquatic resources, the maximum percentage of habitat addressed by the Fisheries Act in 2005 ranged between 0.011% and 0.12% within FMZs for lakes and rivers, respectively (Table 1, Fig. 5, Table H in S1 File).

## Discussion

Fisheries exploitation and aquatic habitat degradation are increasingly becoming recognized across North America and Europe as two main threats to sustainable fish populations and fish species conservation in inland, recreational fisheries [3,4,9]. From a management perspective, a regional understanding of the state of fish stocks and fish habitat is increasingly valuable, especially as A) additional threats become manifested across broad spatial scales (e.g. climate change; [27]), B) economic austerity within governments restricts the number of individual case studies that can be repeatedly surveyed over time (e.g. such as Quigley and Harper’s 2006 audits; [55]), C) fisheries managers understand that anglers often view their choices in fishing spots across broad landscapes (e.g. Ontario—[26]), and D) researchers attempt to contrast and compare the state of the environment among jurisdictions with different management regimes (e.g. Europe—[3]). Here we present a method based primarily at the scale of a tertiary watershed, and presented at the scale of management zones. In Ontario, our method provides estimates for rates of fisheries exploitation and fish habitat protection, as well as valuable insights into key drivers of fish mortality and habitat loss. Our case study confirms some commonly held, but typically difficult to quantify, beliefs on the state of inland fisheries management across temperate ecosystems. Here we discuss the verification of our estimates, some insights into recreational fishery management, how to extend the method further, and the limitations of the approach.

### Productivity and Exploitation

Under the angler harvest (including hooking mortality), it appears that fish populations in southern Ontario were highly exploited in 2005, and are vulnerable to collapse. The model’s fisheries yields rely heavily on existing empirical relationships among fish production, standing fish biomass and environmental conditions. The first we presented is the global relationship between climate and the morpho-edaphic index with a lake commercial fisheries yield (Schlesinger and Regier 1982). On a unit-area basis our yield estimates excluding the Great Lakes were similar to the range of values found in individual lakes in northern [24] and southern Ontario [15]. However, we recognized that Schlesinger and Regier’s 1982 relationship was based on commercial yields, and though it is not known whether recreational yields differ systematically, they are thought to be negatively related to lake sizes [57], which could lead to lower fishable production available to anglers in the Great Lakes. The efficiency of catching fish between recreational and commercial gear might also explain why small yields were generally overestimated compared to recreational creel data (Figure B in S1 File). Alternatively, the theoretical yield of the MEI is expected to be higher than our estimate for smaller bodied fish species that inhabit littoral zones (e.g. Yellow Perch, *Perca flavescens*, Percidae), which contribute a large portion of lake catches in southern Ontario [47] and at 55% of the catch likely accounts for FMZ 17’s low fishable production surplus. Aside from the natural yield, our fishable production estimates didn’t include fish stocking programs, which are found in some southern Ontario lakes (e.g. Lake Simcoe), and are common across North America and Europe in the form of “put-and-take” or "put-grow-take" fisheries [10,58]. While likely not necessary for the purpose of this case study, including stocking numbers would be a simple extension of the model, and easiest to include at the stage of accounting for the angler’s harvest.

On a unit area basis, our river productivity estimates were on average 1.6x higher than lake productivity which matches the roughly 2x empirical expectations found globally [46], and was within the ranges found in southern Ontario warmwater streams [59], and North American salmonid streams in British Columbia [60], Newfoundland [61] and New York [62]. The river estimates were calculated using a different methodology than the lake estimates, yet appear to be in the proper range of relative values, which increases our confidence in the general appropriateness of our river and lake models. Our simplifying assumption that rivers are fully accessible to anglers if they were crossed by a road would be violated if there are barriers to fish movement upstream or downstream of the road crossing, however for all but 1 of the 8 northern FMZs over 85% of the angling effort was directed on lakes and so our accessibility estimates on rivers had a low influence on the estimated surplus fishable production.

The main caveats to our Ontario-specific model do not suggest that there lies a consistent bias in our findings, and so the low rates of surplus fishable production we found confirms concerns that invisible collapses could occur in some Ontario fisheries [12]. Our finding of higher exploitation levels near populated areas also confirms Ontario’s provincial monitoring program’s estimates of regional fishing mortality rates for Walleye and Northern Pike (pers. comm. Dr. Nigel Lester, MNRF), as well as supports the findings of other targeted studies conducted elsewhere in Canada [63]. The agreement with mortality rates from MNRF’s broadscale monitoring program also strengthens our confidence in the catch and harvest estimates used from the Angler Survey. A more general insight from our case study is that hooking mortality has a large influence on the exploitation rates. Across all the FMZs the angler’s catch was about 5 times larger than their harvest [47]. Thus even a low hooking mortality of 20% would double exploitation rates (we modeled a conservative 10% mortality), and be immune to harvest based regulations. Further we noted that angler access was a more important influence in the north, where vast landscapes of lakes and rivers are not easily accessible. Ontario anglers drive an average of 225 km and up to 2659 km per trip along Ontario’s paved and unpaved road networks [25] and apply considerable pressure to accessible fish stocks (e.g. 77% declines in Lake Trout, *Salvelinus namaycush* Salmonidae, populations once lakes open up—[64]). These two insights have direct applicability to future management decisions, and are likely equally important in other temperate recreational fisheries.

### Habitat Protection

The potential rate of impact to fish habitat by human developments within the accessible recreational fisheries in 2005 were highest for rivers in Southern Ontario (0.01% to 0.12%), intermediate for lakes in the Kawartha Lakes FMZ (0.01%) and generally much smaller in the north and on the Great Lakes. The maximum rates were comparable to annual rates of deforestation in northern and southern Ontario in the early 2000s (0.02% and 0.06%, respectively), as well as in the Canadian provinces of Alberta (0.25%) and British Columbia (0.49%) [65]. However, other studies on specific waterbodies generally gave higher rates including 1.9% for the Nairn Creek watershed in Southern Ontario over a 20 year period [66], and 2.5% for wetland degradation in the United States in the early 1980s [67]. Overall, we expected our estimates to be conservative because 1) our frequency distributions of habitat disturbance did not include indirect or cumulative impacts around actual project footprints [68], 2) we did not consider the enforcement wing of the *Fisheries Act* which deters illegal activities, and 3) we did not include projects that were rejected and would have been otherwise built, although they are expected to be low in frequency [69]. However, even a small rate, if unregulated could cumulatively exert significant pressure on Ontario’s aquatic resources. Once habitat is lost it typically has little chance of being replaced, and so on each subsequent year its lost contribution to fisheries production will be subtracted against the baseline yields. In this scenario, cumulative effects follow a power law and grow quickly. Even if only our smallest regional rates of habitat protection were expected to accumulate over 30 years, the area protected would grow to 3% of the total aquatic habitat in the province (including inaccessible lakes and rivers). Note that this rate only represents the physical loss of fish habitat through developments, and not chronic losses through other indirect and non-point sources of pollution.

We have used federal legislation to estimate the amount of fish habitat that was protected in 2005. However, as demonstrated in the few environmental audits available [53–56], the goal of a no-net-loss of fish habitat was likely rarely achieved across Canada. For example, in one audit approximately 63% of the projects surveyed failed to protect or compensate for about 30% of the impact area [55]. In the same study the authors found that actual losses to fish habitat were often double what was permitted within the federal approvals. A set of Canadian [53,56,70,71] and international [36] case studies have generally concluded that to achieve no-net-loss in fish habitat, ratios of compensated habitat to lost fish habitat must account for natural variation, restoration design failure and time-lags at a minimum of roughly 2:1, 4:1, and 8:1, respectively. Thus, although our rate of habitat protection seems small, the actual rate of habitat loss could be much larger. Applying these ratios directly to our datasets would certainly raise our estimates by at least an order of magnitude. We did not attempt a more precise estimate here because in the mid-2000s Canadian developers began applying 2:1 or 3:1 compensation ratios by convention [71], and so more information from the federal regulators or additional statistical sampling with new assumptions would be required.

### Policy Insights

Our findings suggest that although small project which individually impact less than a hectare of aquatic habitat collectively comprise more than 75% of the total number of projects, they barely make up 10% of total predicted habitat impacts (see Fig. 4). From this perspective alone, it would be most effective to focus regulatory, management and scientific resources on minimizing the impacts from large projects, and expend less effort on the smaller projects. This is the apparent approach of the Canadian government which rewrote the *Fisheries Act* in 2012 to no longer directly administer small developments (e.g. docks, culverts), and instead provide self-assessment guidelines for the public [72]. As the European Union and the United States develop their fish habitat management regulations in a time of economic recovery, they may also choose to limit expenses in a similar manner. However, we note that other recent federal legislative changes in Canada imposed mandatory time limits on the regulatory review of large projects even though there was no evidence that the regulatory process had previously been inefficient [51]. Thus artificial regulatory time limits could impede robust critiques of environmental assessments and contribute to poor assessments and restoration design failures [54]. Calls for greater regulatory expediency exist for all governments [51] and so we caution policy-makers to carefully balance a focus on large projects with artificial time-limits on regulatory review. Further, the loss of the reporting of small and medium sized projects would only further exacerbate the knowledge gaps in our understanding of rates of habitat degradation in Canada specifically [29] and more generally across Europe and the United States [5,17]. Comprehensive broad-scale monitoring programs should thus be an integral component of new policy that involves any reductions in regulatory oversight.

On the other hand, the cumulative effects of small and medium sized projects in southern Ontario could be more harmful to the value of the recreational fisheries than large projects in the north. In area alone the largest cumulative impacts were expected to occur in FMZs 16 (south Ontario) and 11 (central Ontario). Both these management zones were estimated to exhibit negative surplus fishable production in this study, were worth CDN$ 172 million and CDN$ 123 million to the Canadian economy in 2005 [47], respectively, and already incurred a high stress index from a legacy of human development [16]. Northern FMZs were worth less on average (~CDN$ 100 million) but contained abundant fishable production, low stress indices and additional fish populations not yet accessible to the fishery. Therefore, in management zones with fragile fisheries, additional stresses could be much more economically harmful if ecological thresholds were surpassed leading to rapid population declines [73,74]. From this perspective, it would be most beneficial to focus regulatory efforts on the management of all southern Ontario developments, no matter what the size, in management zones where recreational fisheries are over-exploited.

### Model Extensions

Recent reviews on the state of global recreational fisheries have identified some common management problems including: large differences in spatial scale between different aspects of fisheries management such as joint community-federal state cooperative regimes [3]; a lack of an integrated/ecosystem approach to fisheries management [4]; few socio-economic models that accurately estimate the value of the fishery [20,22]; little understanding of the combined role of habitat degradation with high exploitation on fish populations [75], and; the management of diffuse stocks based on a little knowledge and vulnerable to the shifting baseline [3,4,10]. Further, Arlinghaus [3] extended Ray Hilborn and colleagues’ three primary influences to fisheries management success to recreational fisheries: 1) spatial scale of management, 2) access to fisheries resources, and 3) the decision making structure of managers. The method we have presented here attempts to address these concerns and primary influences by creating a spatially explicit approach that can be insightful on its own, or extended into new questions. With advances in GIS and satellite imagery, obtaining an inventory of local physical aquatic resources is becoming easier and can be found for other jurisdictions such as the USA [76] and Europe [77]. Once aquatic resources are delineated, the method presented here can be applied at multiple spatial scales, allowing for meaningful comparisons between federal and local management jurisdictions and policies. In our case study, the provincial management zones for fisheries partition the province of Ontario into 20 parcels, whereas the federal management zone for fish habitat includes 3 more provinces and 2 large northern territories. Often, the greatest challenge is to estimate the baseline fish stocks over a region [3], and then subsequently apply the effects of a legacy of human activities (stress indices), stocking enhancements and fisheries harvests. Rather than inferring changes in stock productivity from a time-series of catch-per-unit-effort or abundance indices, this method attempts to predict stock abundances *a priori* before applying catch data, which is useful for data-poor stocks, and appeared to be a fairly robust indicator for Ontario’s fisheries.

Once the approach has been optimized/parameterized and validated it can be extended to investigate other questions and aid management decisions. For example, the economics of a recreational fishery depends not only on its current worth, but how much of the angler’s income would be spent within the regional economy on other recreational pursuits if the fish stocks collapsed. In recent socio-economic angler surveys from Ontario and England, the strength of the recreational fishery is suggested to motivate tourism [7,47], and so in the event of a collapse, the tourist dollars would most likely be lost from the local economy, and spent on other pursuits elsewhere. In Ontario, American tourists are most common in two northern FMZs, 4 and 5, comprising 78% and 72% of the anglers, respectively [47]. While the north is generally high in surplus fishable production and low in potential losses to fish habitat losses, our estimates suggest that the existing fishery is highly utilized and has little room for additional effort unless roads open up access to new resources (Table 1 and 2). Tourist anglers are in decline in Ontario, as is the general economic value of the fishery, and so if managers wanted to retain revenue from these anglers, our model would suggest targeting efforts in FMZs 4 and 5.

## Conclusion

We present here a relatively simple method to address the pervasive knowledge gaps in the state of recreational fisheries and their habitat around the world [3,4,11]. Using established empirical relationships to estimate fish productivity [13,14], and a growing international access to landscape information [37,76,77], it is possible for management jurisdictions to build simple yet spatially explicit baseline models of their fisheries. Our method does not have to be strictly predictive or quantitative. While our case study led to estimates that were generally confirmed by provincial monitoring programs and published research studies, the insights we gained from them were more useful as a qualitative tool for decision making. In the last few decades the toolbox of models for freshwater fisheries biologists and aquatic scientists has been steadily building [18] along with statistical methods to incorporate qualitative and quantitative data into decision making processes and risk assessments. We hope the approach we present here will add to the toolbox and aid with policy decisions on a valuable natural resource, ecosystem and economy.

## Supporting Information

S1 File Additional methods, figures and tables to support the model. (DOCX)

S1 Dataset Spreadsheet of spatially referenced impacts in 2005 in Ontario, as well as impact distributions. (XLSX)
