# Supplementary material for: Estimating Fish Exploitation and Aquatic Habitat Loss across Diffuse Inland Recreational Fisheries
Source: PLoS One. 2015 Apr 15;10(4):e0121895. doi: 10.1371/journal.pone.0121895 (PMC4398429; doi:10.1371/journal.pone.0121895)
Supplement: S1 File — (DOCX) [file pone.0121895.s001.docx]

Estimating fish exploitation and aquatic habitat loss across diffuse inland recreational fisheries

Derrick Tupper de Kerckhove, Charles Kenneth Minns, Cindy Chu

**Supporting Information S1, Additional Methods, Tables and Figures**

Additional details on the methodology and results for obtaining lake and river productivity estimates, and rates of annual and cumulative habitat area protection estimates is provided below and includes summary tables of the results and models used. Please refer as well to the companion Supplemental Materials Microsoft Excel file which contains raw data from the Fisheries and Oceans Canada PATH system database, and environmental audits.

*River Productivity Estimates*

The stepwise multiple linear regression model was built with ten southern watersheds that contained at least five years of data and validated with another twelve southern watersheds using root mean square error as a measure of model performance. For six northern FMZs only two years of data was available but to ensure the regression model accurately represented northern conditions, four of the watersheds were added to the model building dataset and two watersheds were added to the model validation dataset. Thus using the most parsimonious regression model (see model in Table S3), average biomass densities were estimated for each tertiary watershed in Ontario.

*Accessible Fishery Estimates*

Our result confirmed the findings of extensive aerial angler surveys over 500 lakes and gill netting surveys on 65 northern Ontario lakes to demonstrate that 1) northern lake trout abundances were generally 47% lower in exploited lakes, and 2) road access decreased lake trout populations by 77% (Kaufman *et al.* 2009). Further, another study identified that forestry roads initiated angler activity and shoreline development on remote lakes and recommended road closures for proactive fisheries management (Hunt and Lester 2009). Our simplifying assumption that rivers are fully accessible to anglers if they were crossed by a road is violated if there are barriers to fish movement upstream or downstream of the road crossing. While instream barriers are common in Southern Ontario, the road density is very high and so access above and below the barriers is likely usually available within tertiary watersheds. In Northern Ontario the opposite is true where fewer instream barriers and roads are expected to be found.

*Habitat Protection Rate Estimates*

Twenty one data sets of different types of developments on either lakes or rivers were discovered in four audits of the effectiveness of the *Fisheries Act (1985)* (Lange *et al.* 2001, Harper and Quigley 2005, Quigley and Harper 2006, MacNeil *et al.* 2008). Fourteen actual impact assessment values were revealed by DFO from the 2005 dataset to gauge the effectiveness of the datasets we collected. All but two values fell well within the 95% confidence intervals around the mean of the datasets: 1) a road construction project was 100 m^2^ less than our minimum value, and 2) a hydroelectric project was 1.46 x 10^6^ m^2^ larger than our maximum value. While the latter of these two outliers may seem fairly extreme, frequency distributions of human developments are usually biased towards larger values thus high but rare values were still included in our project specific distributions. Importantly, there was no evidence of any systematic biases in our distributions. From the available datasets twenty distribution functions of the area of habitat loss associated with a particular activity were modelled of which all were either log-normal or pareto IV (Table S5).

As mentioned in the discussion of the main text, this rate of habitat protection is an ideal rate because it does not take into account the effectiveness of the *Fisheries Act (1985)* in achieving the goal of no-net-loss of fish habitat in Canada. There are not any environmental audits that explicitly assess the effectiveness of mitigating harm to fish habitat through the Letters of Advice or Operational Statements, however there are three audits that review HADDs to determine the effectiveness of habitat compensation (Lange *et al.* 2001, Quigley and Harper 2006, MacNeil *et al.* 2008), and broader studies on the effectiveness of offset plans in environmental remediation (Minns 2006, Moilanen *et al.* 2009). These studies all suggest that generally habitat impacts are under-estimated during environmental assessments, and the efficacies of compensation habitats are over-estimated. Some of these studies suggest ratios that can be applied to HADDs to account for their uncertainty. However, we chose not to apply any ratios for the following reasons:

1. The objective of the study was to evaluate the intended value of fish habitat protection and therefore the legislation was evaluated at its theoretical potential to protect fish habitat.
2. The uncertainty in the offsets is driven in a large part by project delays and knowledge gaps on effective habitat and assessment restoration techniques (Minns 2006, Quigley and Harper 2006, Moilanen *et al.* 2009). Over the last 30 years these knowledge gaps have been increasingly addressed (Minns *et al.* 2011) and included in new restoration designs. Therefore overtime the rates of uncertainty in HADD and offset sizes should be decreasing yet the rate of this change is not well known. The variation in estimated versus actual impacts sizes can be extremely large (e.g. actual HADD sizes can range up to 8 times the assessed size, and as high as 95 times when including time lags (Moilanen *et al*. 2009), yet in some cases the HADD sizes are larger than the actual impact sizes (Quigley and Harper 2006)). Estimating changes in the efficacy of the law would therefore contribute substantial variation to our model and to be addressed properly should require a separate study.
3. Applying a correction to our estimated rates of habitat protection may not be necessary in all cases because some of the HADD sizes used to create the project specific impact distributions were from environmental audits which conducted independent surveys to verify HADD areas.
4. The project-specific impact area distributions are broad enough that at least some of the uncertainty in the actual HADD sizes will be addressed by randomly sampling from the distribution. Further, our use of the lower and upper bounds of the estimate based on the size of the project that a Letter of Advice represents covers a reasonable range in rates of fish habitat protection, and thus likely addresses even more of the variation in actual HADD sizes and offset ratios.
5. As mentioned in the text, developers began to adopt higher compensation ratios by convention by 2005, and so some of these uncertainties may have already been addressed in No-Net-Loss calculations.

**References**

Harper, D.J. and Quigley, J.T. (2005) No net loss of fish habitat: a review and analysis of habitat compensation in Canada. *Environmental Management* **36**, 343–55.

Hunt, L.M., and Lester, N. (2009) The effect of forestry roads on access to remote fishing lakes in northern Ontario, Canada. *North American Journal of Fisheries Management* **29**: 586–597

Kaufman, S.D., Snucins, E., Gunn, J.M. and Selinger, W. (2009) Impacts of road access on lake trout (*Salvelinus namaycush*) populations: regional scale effects of overexploitation and the introduction of smallmouth bass (*Micropterus dolomieu*). *Canadian Journal of Fisheries and Aquatic Sciences* **66**, 212–223.

Lange, M., Cudmore-Vokey, B.C. and Minns, C.K. (2001) Habitat compensation case study analysis. *Canadian Manuscript Report of Fisheries and Aquatic Sciences* **2576**, 39 p.

MacNeil, J.E., Murphy, S., Ming, D. and Minns, C.K. (2008) Analysis of infilling projects affecting fish habitat in the Great Lakes (1997-2001). *Canadian Manuscript Report of Fisheries and Aquatic Sciences* **2840**, 194 p.

Minns, C.K. (2006) Compensation ratios needed to offset timing effects of losses and gains and achieve no net loss of productive capacity of fish habitat. *Canadian Journal of Fisheries and Aquatic Sciences* **63**, 1172–1182.

Minns, C.K., Randall, R.G., Smokorowski, K.E., et al. (2011) Direct and indirect estimates of the productive capacity of fish habitat under Canada’s Policy for the Management of Fish Habitat: where have we been, where are we now, and where are we going? *Canadian Journal of Fisheries and Aquatic Sciences* **68**, 2204–2227.

Moilanen, A., van Teeffelen, A.J. A., Ben-Haim, Y. and Ferrier, S. (2009) How much compensation is enough? A framework for incorporating uncertainty and time discounting when calculating offset ratios for impacted habitat. *Restoration Ecology* **17**, 470–478.

Quigley, J.T. and Harper, D.J. (2006) Effectiveness of fish habitat compensation in Canada in achieving no net loss. *Environmental management* **37**, 351–66.

Table A. Summary of climate and land use in the tertiary watersheds throughout Ontario.

| **Climate and land use characteristics** | **Minimum** | **Mean** | **Maximum** |
| --- | --- | --- | --- |
| Maximum air temperature (°C) | 18.0 | 24.3 | 28.4 |
| Mean annual air temperature (°C)* | -3.3 | 2.1 | 8.8 |
| Total precipitation (mm) | 484 | 778 | 1097 |
| Growing degree days (above 5°C) | 910 | 1427 | 2357 |
| **Proportion of different land cover types** |  |  |  |
| Water | 0 | 0.08 | 0.35 |
| Settlement and Developed Land* | 0 | 0.01 | 0.33 |
| Cropland | 0.00 | 0.11 | 0.87 |
| Pasture and Abandoned Fields | 0.00 | 0.03 | 0.42 |
| Dense Deciduous Forest* | 0.00 | 0.08 | 0.38 |
| Treed Bog | 0.00 | 0.05 | 0.32 |
| Dense Coniferous Forest | 0.00 | 0.12 | 0.39 |
| Mixed Forest – mainly Deciduous* | 0.00 | 0.08 | 0.29 |
| Mixed Forest – mainly Coniferous* | 0.00 | 0.10 | 0.41 |
| Sparse Coniferous Forest | 0.00 | 0.08 | 0.41 |
| Recent Cutovers | 0.00 | 0.03 | 0.18 |
| Treed Fen | 0.00 | 0.04 | 0.40 |
| Open Bog | 0.00 | 0.04 | 0.38 |
| Recent Burns | 0.00 | 0.01 | 0.13 |
| Old Cuts and Burns | 0.00 | 0.03 | 0.28 |

*indicates variables for the regression model

Table B. Summary of the average number of rivers and the number of years they have been sampled for fish biomass densities (kg⋅ha^-1^) data in the tertiary watersheds of Ontario.

| **Average number of rivers sampled year** | **Number of years with data** | **Tertiary watershed code** | **General location** |
| --- | --- | --- | --- |
| 2.0 | 1 | 2AC | near Wawa Ontario |
| 1.0 | 1 | 2AE | near Wawa Ontario |
| 1.0 | 1 | 2BA | near Wawa Ontario |
| 2.0 | 2 | 2BD | near Wawa Ontario |
| 2.0 | 1 | 2BE | near Wawa Ontario |
| 2.0 | 1 | 2BF | near Wawa Ontario |
| 2.0 | 11 | 2EB | near Lake Simcoe |
| 1.0 | 3 | 2EC | near Lake Simcoe |
| 6.7 | 11 | 2ED | near Lake Simcoe |
| 1.3 | 4 | 2FA | Bruce Peninsula |
| 3.4 | 5 | 2FB | Bruce Peninsula |
| 2.4 | 5 | 2FC | Bruce Peninsula |
| 1.7 | 3 | 2FD | Bruce Peninsula |
| 3.7 | 3 | 2FE | Bruce Peninsula |
| 6.0 | 1 | 2FF | Bruce Peninsula |
| 3.5 | 6 | 2GA | Greater Toronto Area |
| 1.7 | 3 | 2GB | Greater Toronto Area |
| 5.8 | 9 | 2GC | Greater Toronto Area |
| 6.0 | 1 | 2GD | Greater Toronto Area |
| 1.0 | 1 | 2GE | Greater Toronto Area |
| 4.0 | 1 | 2GF | Greater Toronto Area |
| 1.5 | 2 | 2GG | Greater Toronto Area |
| 4.3 | 12 | 2HB | Greater Toronto Area |
| 9.4 | 11 | 2HC | Greater Toronto Area |
| 12.6 | 12 | 2HD | Greater Toronto Area |
| 1.0 | 2 | 2HH | Kawartha Region |
| 1.5 | 5 | 2HJ | Kawartha Region |
| 2.0 | 4 | 2HK | Kawartha Region |

Table C. Results of stepwise multiple regression to predict riverine fish biomass productivity (kg⋅ha^-1^⋅yr^-1^) from climate and landscape variables in the surrounding tertiary watersheds.

| N: 14 Multiple R: 0.874 Squared multiple R: 0.765 | | | |  |  |
| --- | --- | --- | --- | --- | --- |
|  |  |  |  |  |  |
| Adjusted squared multiple R: 0.722  Standard error of estimate: 17.862 | | | |  |  |
|  |  |  |  |  |  |
| Effect | Coefficient | Std Error | Std Coefficient | t | P(2 Tail) |
| Constant | 63.166 | 22.979 | 0 | 2.7 | 0.019 |
| Settlement and developed land | -159.813 | 69.946 | -0.402 | -2.285 | 0.043 |
| Mean annual air temperature | 7.925 | 2.381 | 0.585 | 3.328 | 0.007 |
|  |  |  |  |  |  |
| Analysis of Variance | |  |  |  |  |
|  |  |  |  |  |  |
| Source | Sum-of-Squares | df | Mean-Square | F-ratio | P |
| Regression | 11393.691 | 2 | 5696.845 | 17.856 | 0 |
| Residual | 3509.417 | 11 | 319.038 |  |  |

Table D. Fish species commonly targeted by anglers in Ontario, and their average weights as harvested by anglers and recorded in provincial creel databases.

| **Common name** | **Scientific name** | **Weight (gr)** |
| --- | --- | --- |
| Panfish species | Centrarchidae | 155 |
| northern pike | *Esox lucius* (Esocidae) | 1719 |
| Muskellunge | *Esox masquinongy* (Esocidae) | 1450* |
| channel catfish | *Ictalurus punctatus* (Ictaluridae) | 1450* |
| smallmouth bass | *Micropterus dolomieu* (Centrarchidae) | 544 |
| largemouth bass | *Micropterus salmoides* (Centrarchidae) | 681 |
| coho salmon | *Oncorhynchus kisutch* (Salmonidae) | 3000** |
| rainbow trout | *Oncorhynchus mykiss* (Salmonidae) | 1350 |
| chinook salmon | *Oncorhynchus tshawytscha* (Salmonidae) | 3000** |
| yellow perch | *Perca flavescens* (Percidae) | 126 |
| atlantic salmon | *Salmo salar* (Salmonidae) | 3000** |
| brown trout | *Salmo trutta* (Salmonidae) | 3000** |
| brook trout | *Salvelinus fontinalis* (Salmonidae) | 343 |
| lake trout | *Salvelinus namaycush* (Salmonidae) | 1084 |
| Walleye | *Sander vitreus* (Percidae) | 748 |

* General weight for sportfish identified in the 2005 angler surveys but not within creel. These species make up 13% of the catch and 20% of the harvest (by weight) and include generally large species (e.g. muskellunge) because panfish were already estimated.

** Estimate for large salmonids that were not included in the creel studies taken as an average from US and Ontario angling fact sheets and recreational fishing magazines. These species make up 3% of the catch and 5% of the harvest (by weight), and are generally caught in the Great Lakes FMZs (70% versus all FMZs).

Table E. Frequency distributions of the amount of habitat lost for ecosystem and project type used to estimate habitat protection rates in Ontario in 2005. Note that a project type may be described by multiple models which address different works. For full breakdown of the models used for the 2005 impacts please see online database.

| **Model Name** | **Ecosystem** | **Project Types** | **Distribution** | **Parameters** |
| --- | --- | --- | --- | --- |
| General Large HADD | All Types | Contaminated Site Remediation, Unidentified Impacts | Pareto IV | Scale: e^11^,  Ineq: e^0.5^,  Shape: e^0.8^ |
| General Letters of Advice | All Types | Control Nuisance Species, Log Handling, Instream Works, Shoreline Works, Structures in Water, Water Management, Watercourse Crossings | Pareto IV | Scale: e^4^,  Ineq: e^-1.5^,  Shape: e^0.1^ |
| General Marina | All Types | Structures in Water | Log Normal | Mean: 8.2,  SD: 0.9 |
| General Dock | All Types | Structures in Water | Log Normal | Mean: 4.0,  SD: 1.0 |
| General Dredge | All Types | Dredging | Log Normal | Mean: 5.9,  SD: 2.0 |
| General Road | All Types | Watercourse Crossings | Log Normal | Mean: 6.7,  SD: 1.1 |
| General Water Management | All Types | Water Management | Log Normal | Mean: 6.7,  SD: 1.7 |
| General Breakwater | All Types | Shoreline Works and Structures in Water | Log Normal | Mean: 5.3  SD: 1.8 |
| General Lake | Lacustrine | Shoreline and Instream Works, Structures in Water | Log Normal | Mean: 5.1,  SD: 1.9 |
| Shoreline Lake | Lacustrine | Shoreline Works (Foreshore, Streambank and Riparian Work), Structures in Water | Log Normal | Mean: 4.8,  SD: 1.6 |
| Mining Lake | Lacustrine | Mineral Aggregate, Oil & Gas Exploration, Extraction, Production | Log Normal | Mean: 10.7,  SD: 1.8 |
| General River | Riverine | Shoreline and Instream Works, Structures in Water, Watercourse Crossings | Log Normal | Mean: 5.0,  SD: 2.1 |
| Shoreline River | Riverine | Shoreline Works (Foreshore, Streambank and Riparian Work), Structures in Water | Log Normal | Mean: 4.8,  SD: 1.9 |
| Mining River | Riverine | Mineral Aggregate, Oil & Gas Exploration, Extraction, Production | Log Normal | Mean: 6.8,  SD: 1.8 |
| Hydroelectric River | Riverine | Water Management | Log Normal | Mean: 9.8,  SD: 2.0 |
| Channel Alignment River | Riverine | Instream Works, Water Management | Log Normal | Mean: 7.2,  SD: 1.4 |
| Channel Crossing River | Riverine | Watercourse Crossings | Log Normal | Mean: 5.4,  SD: 1.3 |

Table F. Lake fishable production in Fisheries Management Zones (FMZ) throughout Ontario with and without the stress index applied to the climate-MEI estimates.

| FMZ | FMZ Area | Lake Area | Fishable Production | | |
| --- | --- | --- | --- | --- | --- |
|  |  |  | Pristine | Stress Index | With Stress |
|  | (km2) | (km2) | (t/yr) |  | (t/yr) |
| 1 | 108457.66 | 4761.28 | 2976.12 | 0.11 | 2655.36 |
| 2 | 220917.83 | 19217.34 | 9919.14 | 0.11 | 8867.39 |
| 3 | 142637.73 | 4885.03 | 3402.34 | 0.11 | 3031.15 |
| 4 | 60472.83 | 11858.43 | 4501.80 | 0.16 | 3779.05 |
| 5 | 44631.30 | 12892.44 | 5137.72 | 0.21 | 4036.41 |
| 6 | 43609.59 | 6974.34 | 1762.07 | 0.26 | 1298.08 |
| 7 | 61897.69 | 2643.89 | 1392.47 | 0.21 | 1093.23 |
| 8 | 102665.68 | 3224.78 | 1843.18 | 0.23 | 1427.69 |
| 9 | 29035.24 | 29035.24 | 5711.71 | 0.26 | 4213.04 |
| 10 | 56934.67 | 3361.85 | 1310.35 | 0.27 | 959.59 |
| 11 | 19083.13 | 2246.64 | 984.66 | 0.26 | 727.15 |
| 13 | 17872.26 | 2622.23 | 2213.15 | 0.30 | 1557.65 |
| 14 | 18962.73 | 2774.73 | 2341.86 | 0.30 | 1648.24 |
| 15 | 37847.36 | 1909.20 | 1006.72 | 0.29 | 715.93 |
| 16 | 52477.45 | 1434.01 | 1006.01 | 0.37 | 636.67 |
| 17 | 9376.26 | 370.04 | 378.06 | 0.39 | 230.59 |
| 18 | 25253.11 | 1315.78 | 892.25 | 0.32 | 611.16 |
| 19 | 13693.23 | 13608.56 | 20025.11 | 0.42 | 11555.88 |
| 20 | 11832.55 | 11430.96 | 10591.72 | 0.40 | 6378.31 |

Table G. Riverine fish biomass production (kg⋅ha^-1^⋅yr^-1^) in Fisheries Management Zones (FMZ) throughout Ontario for all fish species and those only targeted by the recreational fishery.

|  | **Production (kg⋅ha^-1^⋅yr^-1^) for all species** | | | **Production (kg⋅ha^-1^⋅yr^-1^) for target species** | | |  |
| --- | --- | --- | --- | --- | --- | --- | --- |
| **FMZ** | Mean | Minimum | Maximum | Mean | Minimum | Maximum | |
| 1 | 44.6 | 37.0 | 51.9 | 26.8 | 22.2 | 31.1 | |
| 2 | 41.3 | 17.9 | 60.0 | 24.8 | 10.7 | 36.0 | |
| 3 | 54.4 | 9.5 | 60.0 | 32.6 | 5.7 | 36.0 | |
| 4 | 42.8 | 20.3 | 58.2 | 25.7 | 12.2 | 34.9 | |
| 5 | 41.4 | 13.3 | 58.2 | 24.9 | 8.0 | 34.9 | |
| 6 | 47.6 | 35.8 | 58.2 | 29.3 | 21.5 | 34.9 | |
| 7 | 31.8 | 8.9 | 53.9 | 18.8 | 5.3 | 32.3 | |
| 8 | 43.7 | 7.0 | 57.7 | 26.2 | 4.0 | 34.6 | |
| 10 | 24.7 | 4.4 | 51.6 | 15.5 | 2.6 | 31.0 | |
| 11 | 38.2 | 26.8 | 86.0 | 22.9 | 16.1 | 51.6 | |
| 15 | 65.8 | 29.6 | 86.0 | 38.4 | 17.8 | 51.6 | |
| 16 | 101.8 | 63.6 | 123.2 | 59.8 | 38.2 | 73.9 | |
| 17 | 71.3 | 41.7 | 106.7 | 45.1 | 25.0 | 57.9 | |
| 18 | 75.4 | 57.6 | 89.7 | 45.2 | 34.6 | 53.8 | |
| **Min** | **24.7** | **4.4** | **51.6** | **15.5** | **2.6** | **31.0** | |
| **Mean** | **50.0** | **24.3** | **70.1** | **30.1** | **14.6** | **41.6** | |
| **Max** | **101.8** | **63.6** | **123.2** | **59.8** | **38.2** | **73.9** | |

Table H. The lower bound of estimates of rates of habitat protection from 2005 applications for development permits under the *Fisheries Act* (*1985*) and the percentage of aquatic habitat accessible by roads in lakes and rivers within Ontario’s Fisheries Management Zones.

| Ontario Fisheries Management Zone | Lake | | | | River | | | |
| --- | --- | --- | --- | --- | --- | --- | --- | --- |
|  | Area (km^2^) | Impact (km^2^) | Access (%) | Habitat Protected (%) | Area (km^2^) | Impact (km^2^) | Access (%) | Habitat Protected (%) |
| 1. Far North - Hudson Bay Lowlands | 4761.28 | 0.00000 | 0.05 | 0.000000 | 1005.69 | 0.00006 | 0.60 | 0.000011 |
| 2. Far North - West | 19217.34 | 0.00008 | 0.19 | 0.000002 | 2291.30 | 0.00025 | 0.85 | 0.000013 |
| 3. Far North - East | 4885.03 | 0.00000 | 0.54 | 0.000000 | 1380.65 | 0.06817 | 0.65 | 0.007634 |
| 4. Red Lake/Sioux Lookout | 11858.43 | 0.01426 | 0.56 | 0.000213 | 1091.50 | 0.00054 | 1.00 | 0.000050 |
| 5. Fort Frances / Lake of the Woods | 12892.44 | 0.00358 | 0.76 | 0.000037 | 409.74 | 0.00079 | 1.00 | 0.000192 |
| 6. Thunder Bay | 6974.34 | 0.00085 | 0.84 | 0.000014 | 425.25 | 0.00217 | 1.00 | 0.000510 |
| 7. Wawa and Nipigon | 2643.89 | 0.00009 | 0.41 | 0.000008 | 661.62 | 0.00203 | 1.00 | 0.000307 |
| 8. Kirkland Lake | 3224.78 | 0.01739 | 0.51 | 0.001060 | 1520.99 | 0.00330 | 0.91 | 0.000240 |
| 9. Lake Superior | 29035.24 | 0.00019 | 1.00 | 0.000001 | 35.36 | 0.00024 | 1.00 | 0.000672 |
| 10. Sudbury/Sault Ste. Marie/Manitoulin I. | 3361.85 | 0.00283 | 0.84 | 0.000100 | 564.56 | 0.01078 | 1.00 | 0.001910 |
| 11. North Bay | 2246.64 | 0.00196 | 0.89 | 0.000098 | 298.86 | 0.08140 | 1.00 | 0.027237 |
| 13. Lake Huron | 2622.23 | 0.00741 | 1.00 | 0.000283 | 0.00 | 0.00000 | 1.00 | 0.000000 |
| 14. Georgian Bay/North Channel | 2774.73 | 0.00945 | 1.00 | 0.000341 | 17.50 | 0.00059 | 1.00 | 0.003394 |
| 15. Bancroft/Algonquin | 1909.20 | 0.01966 | 0.80 | 0.001293 | 366.45 | 0.00981 | 1.00 | 0.002678 |
| 16. Guelph (including Lake Simcoe) | 1434.01 | 0.00553 | 1.00 | 0.000386 | 587.63 | 0.70644 | 1.00 | 0.120218 |
| 17. Kawartha Lakes | 370.04 | 0.04149 | 1.00 | 0.011246 | 166.83 | 0.01940 | 1.00 | 0.011630 |
| 18. Eastern Ontario | 1315.78 | 0.00103 | 0.85 | 0.000092 | 384.60 | 0.07306 | 1.00 | 0.018995 |
| 19. Lake Erie/Lake St. Clair/Lower Niagara R. | 13608.56 | 0.00083 | 1.00 | 0.000006 | 35.54 | 0.00011 | 1.00 | 0.000307 |
| 20. Lake Ontario/St. Lawrence R./Upper Niagara R. | 11430.96 | 0.00100 | 1.00 | 0.000009 | 20.75 | 0.01409 | 1.00 | 0.067887 |


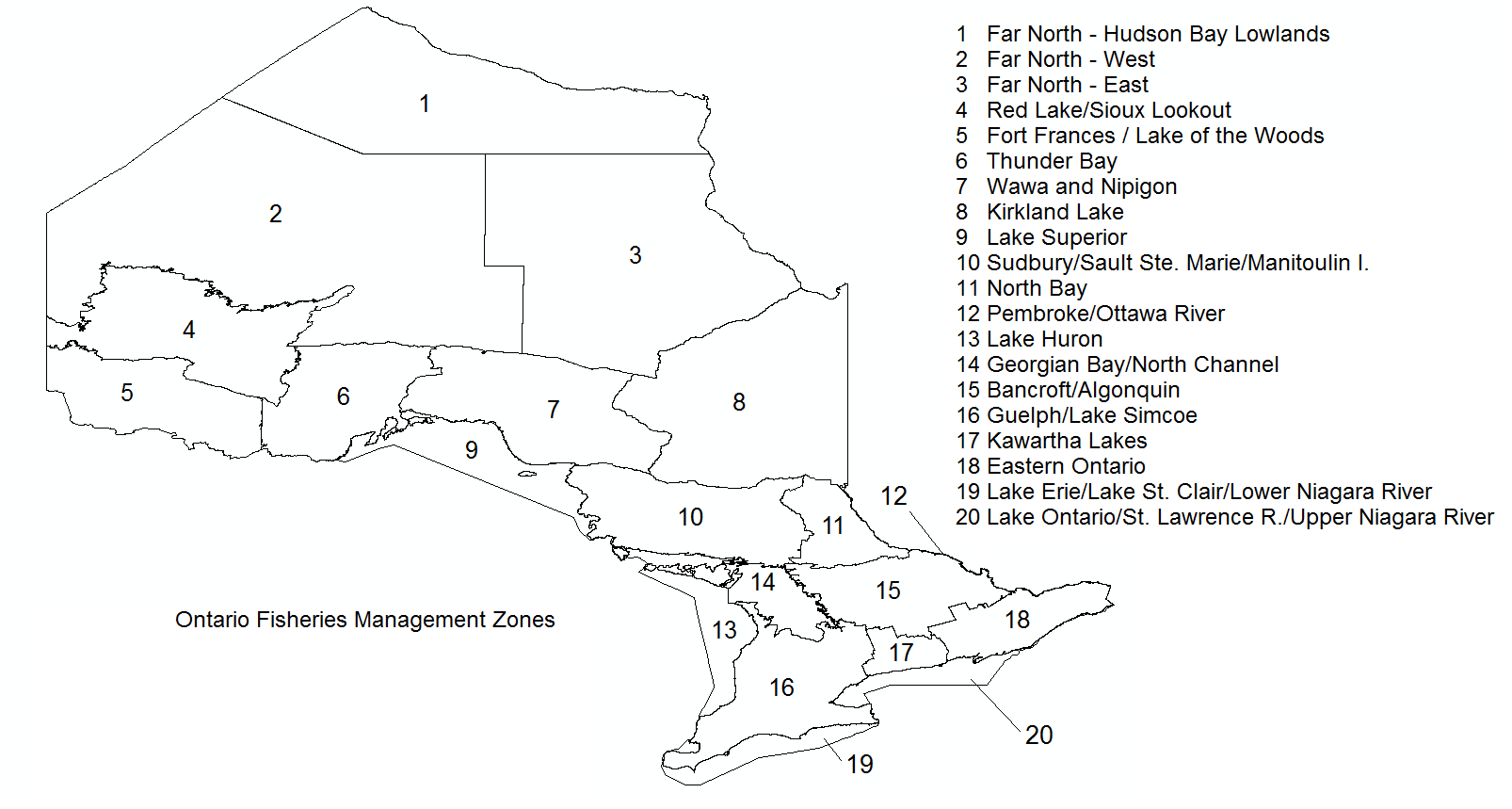


Figure A. Ontario’s Fisheries Management Zones.


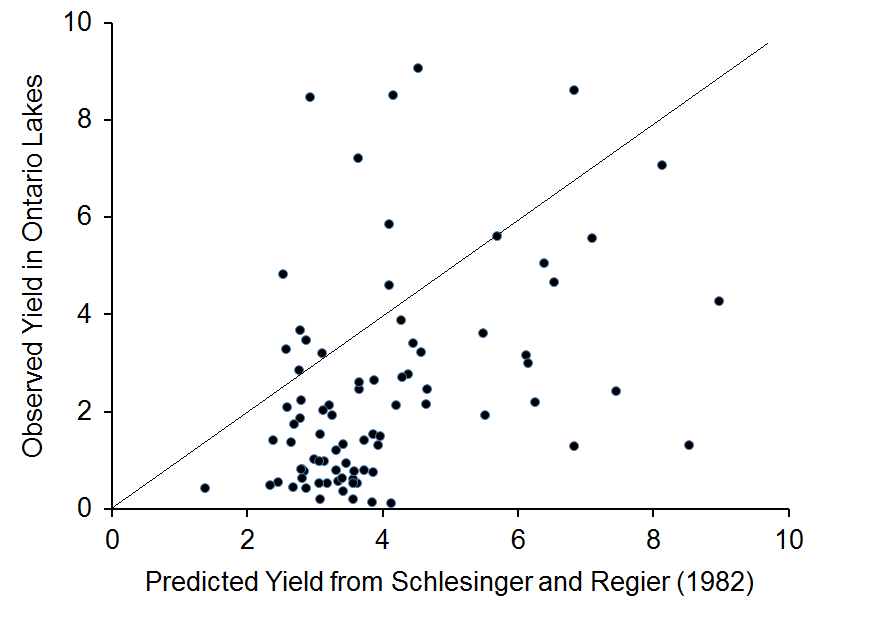


Figure B. The predicted commercial fishery sustainable yield (kg⋅ha^-1^⋅yr^-1^) from a re-analysis of Schlesinger and Regier (1982) data versus actual recreational fisheries yields from across Ontario. The solid line indicates the 1:1 relationship.

Figure C. Observed and predicted fish biomass production (kg⋅ha^-1^⋅yr^-1^) in 14 tertiary watersheds in Ontario. The solid line indicates the 1:1 relationship.
